# Ontogenetic Development of Weberian Ossicles and Hearing Abilities in the African Bullhead Catfish

**DOI:** 10.1371/journal.pone.0018511

**Published:** 2011-04-12

**Authors:** Walter Lechner, Egon Heiss, Thomas Schwaha, Martin Glösmann, Friedrich Ladich

**Affiliations:** 1 Department of Behavioural Biology, University of Vienna, Vienna, Austria; 2 Department of Morphology, University of Vienna, Vienna, Austria; 3 VetImaging Unit, VetCore - Core Facility for Research, University of Veterinary Medicine Vienna, Vienna, Austria; University of Maryland, United States of America

## Abstract

**Background:**

The Weberian apparatus of otophysine fishes facilitates sound transmission from the swimbladder to the inner ear to increase hearing sensitivity. It has been of great interest to biologists since the 19^th^ century. No studies, however, are available on the development of the Weberian ossicles and its effect on the development of hearing in catfishes.

**Methodology/Principal Findings:**

We investigated the development of the Weberian apparatus and auditory sensitivity in the catfish *Lophiobagrus cyclurus*. Specimens from 11.3 mm to 85.5 mm in standard length were studied. Morphology was assessed using sectioning, histology, and X-ray computed tomography, along with 3D reconstruction. Hearing thresholds were measured utilizing the auditory evoked potentials recording technique. Weberian ossicles and interossicular ligaments were fully developed in all stages investigated except in the smallest size group. In the smallest catfish, the intercalarium and the interossicular ligaments were still missing and the tripus was not yet fully developed. Smallest juveniles revealed lowest auditory sensitivity and were unable to detect frequencies higher than 2 or 3 kHz; sensitivity increased in larger specimens by up to 40 dB, and frequency detection up to 6 kHz. In the size groups capable of perceiving frequencies up to 6 kHz, larger individuals had better hearing abilities at low frequencies (0.05–2 kHz), whereas smaller individuals showed better hearing at the highest frequencies (4–6 kHz).

**Conclusions/Significance:**

Our data indicate that the ability of otophysine fish to detect sounds at low levels and high frequencies largely depends on the development of the Weberian apparatus. A significant increase in auditory sensitivity was observed as soon as all Weberian ossicles and interossicular ligaments are present and the chain for transmitting sounds from the swimbladder to the inner ear is complete. This contrasts with findings in another otophysine, the zebrafish, where no threshold changes have been observed.

## Introduction

Otophysine fish comprise four orders (Cypriniformes (carps and relatives), Characiformes (tetras), Gymnotiformes (South American knifefishes) and Siluriformes (catfishes)) containing approximately 8000 species [1]. This makes them the dominant freshwater fish-group worldwide. They possess a highly complex character, the Weberian apparatus, which was first described by Ernst Heinrich Weber in 1820 [2]. It consists of the fused anteriormost vertebrae (“complex vertebra” [3]) and up to four Weberian ossicles which connect the swimbladder to the inner ear. The Weberian ossicles (tripus, intercalarium, scaphium and claustrum – see Dahdul et al. [4] for a review of the different names used by several authors for the ossicles) transmit oscillations of the swimbladder in a sound field to the inner ear and enhance hearing sensitivity [5], [6], [7], [8]. The Weberian apparatus shows a huge morphological variability, in particular within the order Siluriformes (catfishes) (see [9] for a review). Catfishes inhabit all continents but Antarctica [10] and are (with the exception of cypriniforms) the most successful freshwater fish order (about 3100 species). Their hearing abilities depend on the size and number of Weberian ossicles as well as on swimbladder size [11].

The ontogenetic development of Weberian ossicles has been studied in several cypriniforms ([12], [13], [14], [15], i. e., [16], in particular in the zebrafish [17], [18], [19] - see Hoffmann and Britz [20] for an overview of the numerous studies). Further investigations have been conducted in several characiforms [14], [19], and in a few catfish species (in the ariid *Galeichthys felis* (nowadays *Ariopsis felis*) [21], the clariid *Clarias gariepinus* [22], the silurid *Silurus asotus* [23], the bagrid *Pseudobagrus ichikawai* (nowadays *Coreobagrus ichikawai*) [24], the callichthyid *Corydoras paleatus* and the ictalurids *Noturus exilis*, *N. miurus* and *Ictalurus punctatus* [25], [26]). No studies, however, have been conducted on gymnotiforms. The bottom line of these studies is that the Weberian ossicles scaphium, intercalarium and tripus derive from the first, second and third vertebra, respectively, and that no clear information is available on the size or age at which the interossicular ligaments develop. The order of appearance of ossicles seems to follow a general pattern. According to Grande and Young [19], the tripus is the first ossicle to differentiate, followed by the formation of the intercalarium and scaphium; the claustrum is the last to form.

It remains unclear when the Weberian apparatus is completely developed (including interossicular ligaments) and starts to transmit sounds from the swimbladder to the ear.

Numerous morphological studies have been conducted on the ontogenetic development of the auditory periphery in fish, in particular on Weberian ossicles. In contrast, little information is available on the development of hearing in fishes, especially in otophysines. Studies in non-otophysine fishes regularly observed an increase in hearing sensitivity with size (Kenyon [27] in the damselfish *Stegastes partitus*, Iwashita et al. [28] in the Red Sea bream *Pagrus major*, Wysocki and Ladich [29] in the labyrinth fish *Trichopsis vittata*, Higgs et al. [30] in the clupeid *Alosa sapidissima*, Sisneros and Bass [31] in the plainfin midshipman *Porichthys notatus*, and Vasconcelos and Ladich [32] in the Lusitanian toadfish *Halobatrachus didactylus*). Only Egner and Mann [33] found a slight decrease in hearing sensitivity at low frequencies during ontogeny of the damselfish *Abudefduf saxatilis*; Belanger et al. [34] found no differences in hearing abilities of different size stages in the round goby *Neogobius melanostomus*.

Only few studies on the development of hearing have been conducted on otophysines. Popper [35] compared hearing abilities of two size groups of goldfish (5 and 10 cm) and reported no differences in hearing acuity between those groups. In the cyprinid zebrafish *Danio rerio*, Higgs et al. [36] (25–50 mm) and Zeddies and Fay [37] (4 days post-fertilization to adult) used different techniques (AEP versus startle response) but found no differences in the stimulus levels and frequency bandwidth to which fish responded. In a subsequent study using 10–45 mm zebrafish, Higgs et al. [38] again found no changes in the absolute hearing thresholds, but an expansion of maximum detectable frequency from 200 Hz to 4000 Hz. This increase of the range of detectable frequencies was attributed to the development of the Weberian ossicles. In a recent study on catfish, Lechner et al. [39] found a significant frequency-dependent change in hearing thresholds with size in the African mochokid squeaker catfish *Synodontis schoutedeni*, but no change in the range of detectable frequencies.

The present study was designed to investigate the ontogenetic development of hearing abilities in parallel to the development of the auditory periphery (Weberian ossicles) in a representative of the order Siluriformes. We chose the African claroteid catfish *Lophiobagrus cyclurus* and started at earlier stages than in the study on *S. schoutedeni*.

## Results

**Figure 1:**
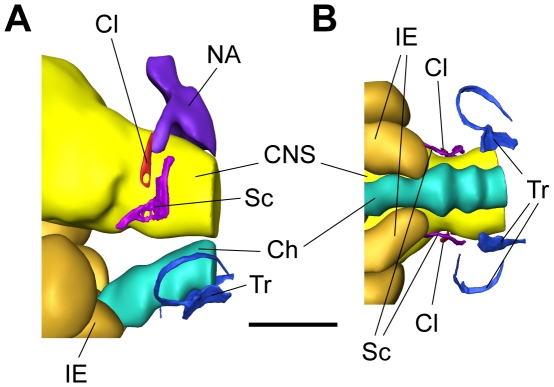
Weberian ossicles and surrounding tissue structures of a specimen of group XS. 3D-reconstruction of the posterior skull region of a 11.3 mm SL specimen of *Lophiobagrus cyclurus* based on serial semithin sections. Weberian ossicles (tripus, scaphium, claustrum), inner ear, parts of CNS, chorda and vertebral column are shown. (A) lateral view, (B) ventral view. Ch – Chorda, Cl – Claustrum, CNS – Central nervous system, IE – Inner ear, NA – Neural arch, Sc – Scaphium, Tr – Tripus, scale bar = 300 µm: anterior is to the left, posterior to the right, (A): dorsal above, ventral below.

**Figure 2:**
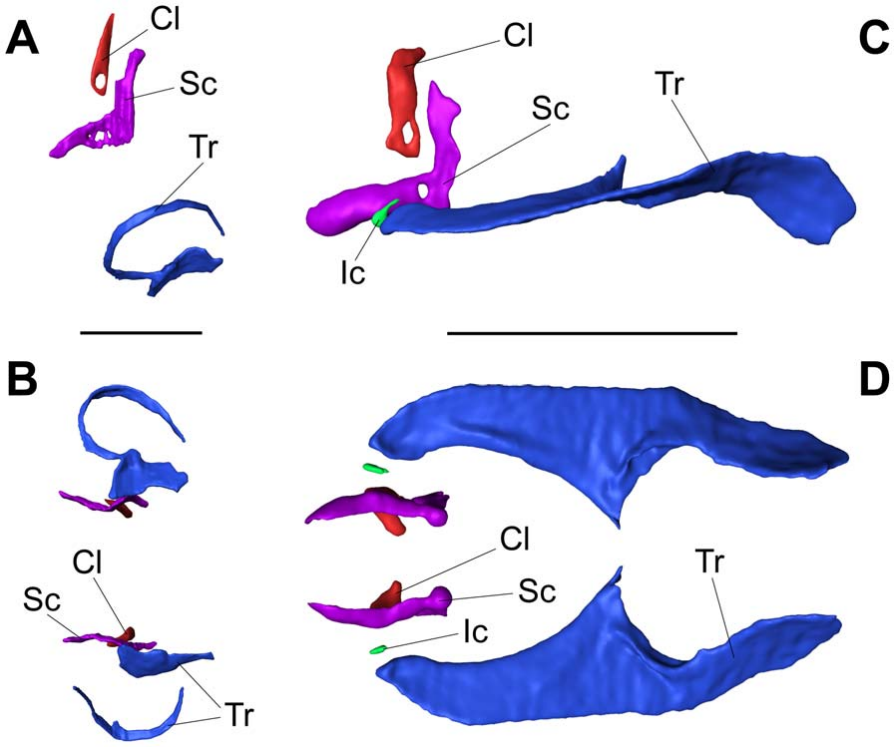
Comparison of Weberian ossicles. 3D-reconstruction based on serial semithin section photomicrographs (A, B) and based on an image stack from a µCT scan (C, D) showing isolated Weberian ossicles. (A) shows a lateral and (B) a ventral view of a specimen of 11.3 mm SL (group XS) and (C) lateral and (D) ventral view of a 85.5 mm SL specimen (group XL). Cl – Claustrum, Ic – Intercalarium, Sc – Scaphium, Tr – Tripus; scale bars in A, B = 300 µm and in C, D = 3 mm; anterior is to the left, posterior to the right, (A), (C): dorsal above, ventral below.

**Figure 3:**
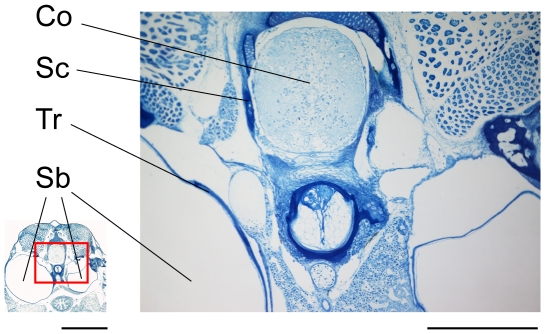
Semithin section in the region of the Weberian ossicles. Near-cross section of a specimen of group XS (SL = 11.3 mm) in the area of the anteriormost vertebrae and the Weberian ossicles. Ossified areas of scaphium and tripus are visible, but there is no indication of interossicular ligaments or intercalarium. Sc – Scaphium, Tr – Tripus, Sb – Swimbladder, Co – Spinal cord; scale bar = 300 µm (right) and 1 mm (left – for overview picture). This is one of the pictures used for 3D reconstructions of group XS (Figure 1, Figure 2 A, B).

**Figure 4:**
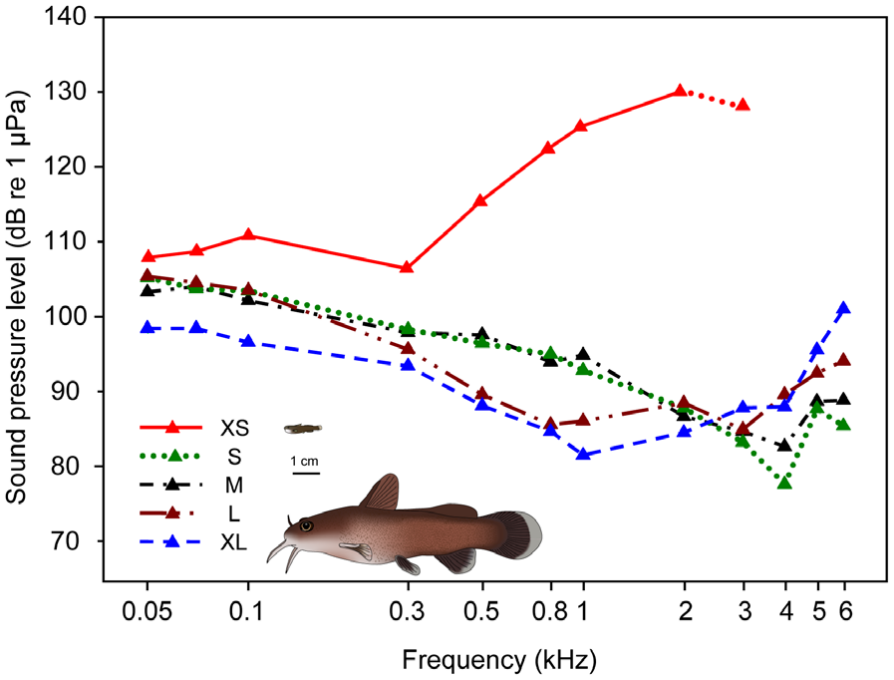
Auditory evoked potential audiograms of the five size groups. Mean hearing thresholds of representatives of size groups XS (N = 7), S (N = 7), M (N = 7), L (N = 8) and XL (N = 7) of *Lophiobagrus cyclurus*. Catfish pictures show representative specimens of group XS (upper) and XL (lower) drawn to scale for comparative purposes.

In the smallest size group XS, the chain of ossicles was not yet fully developed (Figure 1, Figure 2 A, B) and consisted of the claustrum, scaphium and tripus. The tripus on the right was bipartite, whereas the left one was a single ossicle and nearly completely attached to the swimbladder wall (Figure 1, Figure 2 A, B, Figure 3). The scaphium and claustrum were located anterior-dorsally to the tripus right below the first vertebra (Figure 1). The intercalarium as well as the interossicular ligaments were missing (Figure 3) and thus no connection existed between the tripus and the scaphium at this stage (Figure 2 A, B, Figure 3). This not yet fully developed Weberian apparatus, the missing ossicles and interossicular ligaments had major impact on the hearing abilities of group XS. Group XS specimens were less sensitive at all frequencies (Fig. 4, Table 1). Comparison between the audiograms by a two-factor ANOVA revealed significant overall differences between the groups (F_4, 343_ = 174.98, P<0.001) and a significant interaction between group and frequency (F_41, 343_ = 11.54, P<0.001). Thus changes in auditory sensitivity showed different trends at different frequencies. According to Scheffé's post hoc test group XS differed from all other size groups. Thresholds of specimens of group XS rose rapidly above 300 Hz, and only two out of six specimens responded to tone bursts at 3 kHz and none at 4, 5 and 6 kHz. Group XS showed its highest hearing sensitivity at 300 Hz (106 dB re 1 µPa, Figure 4, Table 1).

**Table 1:** Hearing threshold values.

f (kHz)	XS	S	M	L	XL
0.05	107.5±0.89	105.1±0.59	103.3±0.78	105.4±0.91	98.4±0.61
0.07	108.3±1.31	103.7±1.90	104.0±0.53	104.5±0.73	98.4±1.00
0.1	110.5±1.82	103.4±1.76	102.1±0.40	103.5±0.91	96.6±1.19
0.3	106.0±2.16	98.3±1.57	97.9±1.99	95.6±1.79	93.4±1.39
0.5	115.1±0.96	96.4±2.71	97.6±1.43	89.6±2.63	88.1±1.52
0.8	122.3±2.40	95.0±1.91	94.0±2.14	85.6±2.62	84.7±2.69
1	125.3±1.28	92.9±2.53	94.9±1.53	86.1±2.94	81.6±2.06
2	130.1±1.35	87.9±2.57	86.7±1.80	88.5±2.35	84.6±2.38
3	128±6.00	83.3±3.60	84.7±3.41	85.0±2.85	87.9±1.83
4	-	77.7±2.78	82.7±1.89	89.6±2.41	88.0±3.01
5	-	87.7±2.35	88.7±1.97	92.5±1.72	95.6±2.40
6	-	85.4±2.68	88.9±2.15	94.1±1.33	101.0±1.51

Mean hearing threshold values (+/− s.e.m., dB re 1 µPa) of the five size groups of *L. cyclurus* at each frequency tested. f = frequency; for exact size ranges see Materials and Methods.

**Figure 5:**
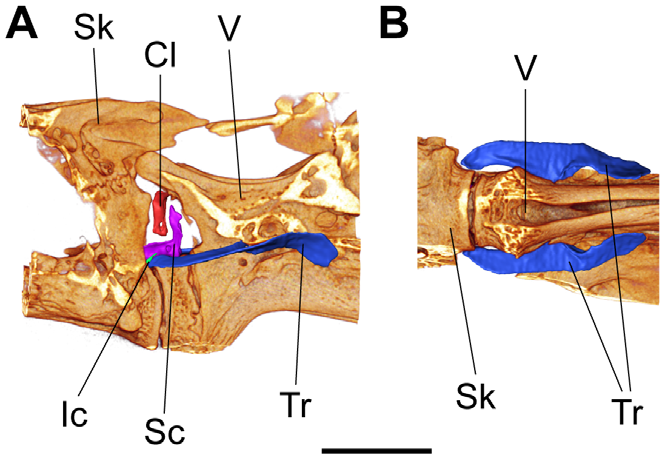
Weberian ossicles and surrounding structures of a specimen of group XL. 3D-reconstruction of the cranial region of the vertebral column of an 85.5 mm SL specimen of *Lophiobagrus cyclurus* based on an image stack from a µCT scan. Weberian ossicles (tripus, intercalarium, scaphium and claustrum) and parts of the skull and vertebral column are shown. (A) lateral view, (B) ventral view. Light regions indicate areas where bony parts (shields formed by transverse processes of the vertebrae and processes of skull bones) are left away to provide a better view of the ossicles. Cl – Claustrum, Ic – Intercalarium, Sc – Scaphium, Sk – Skull, Tr – Tripus, V – Vertebral column; scale bar = 3 mm; anterior is to the left, posterior to the right, (A): dorsal above, ventral below.

**Figure 6:**
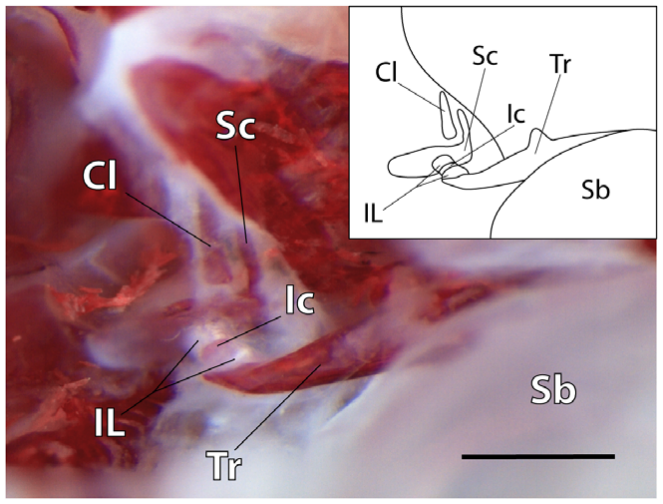
Chain of Weberian ossicles. Photomicrograph and overview drawing of an alizarin-stained specimen of 27.7 mm SL (group S) showing a complete chain of Weberian ossicles and interossicular ligaments. Surrounding bones and tissues have been removed. Cl – Claustrum, Ic – Intercalarium, IL – Interossicular ligaments, Sb – Swimbladder (part), Sc – Scaphium, Tr – Tripus; scale bar = 500 µm; anterior is to the left, posterior to the right, dorsal above, ventral below.

**Figure 7:**
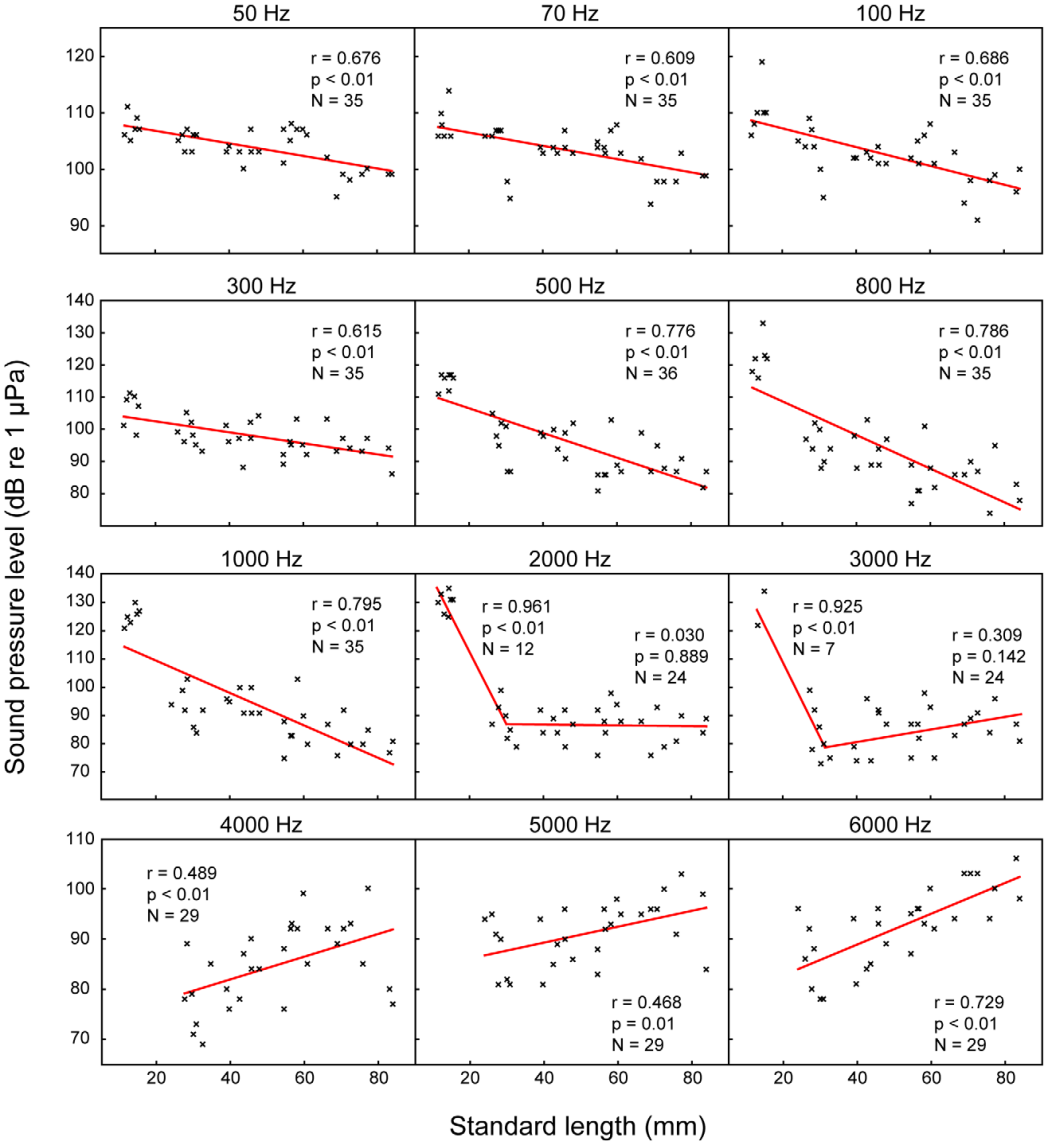
Correlations between auditory thresholds and fish size at each frequency tested. Plots of hearing thresholds of each individual against standard-length at each frequency tested. N-values, Pearson's correlation coefficients and significances are given in graphs. Regression equations: x = standard length, y = hearing threshold (dB re 1 µPa); 50 Hz: y = −0.11 x+108.90; 70 Hz: y = −0.12 x+108.92; 100 Hz: y = −0.17 x+110.51; 300 Hz: y = −0.17 x+105.66; 500 Hz: y = −0.38 x+114.09; 800 Hz: y = −0.52 x+118.88; 1000 Hz: y = −0.57 x+120.92; 2000 Hz: breaking point (BP) = 30.18 mm, SL<BP: y = −2.62 x+164.96, SL>BP: y = 0.01 x+85.64; 3000 Hz: BP = 30.77 mm, SL<BP: y = −2.88 x+167.74, SL>BP: y = 0.15 x+76.68; 4000 Hz: y = 0.23 x+72.87; 5000 Hz: y = 0.16 x+83.01; 6000 Hz: y = 0.31 x+76.61. Regression lines in 2000 Hz and 3000 Hz were drawn according to the results of the segmented linear regression calculation. Note two p and r values (one for each regression) in graphs of 2000 Hz and 3000 Hz.

In contrast, specimens of size groups S to XL possessed a well-developed chain of Weberian ossicles consisting of the tripus, intercalarium, scaphium, claustrum and of interossicular ligaments (Figure 5, Figure 2 C, D, Figure 6). The intercalarium of L. cyclurus showed only a slight indication of an ascending processus (an ascending processus of the intercalarium is a typical characteristic for the intercalaria of many basal otophysans). As an obvious consequence of ossicular development all specimens of groups S – XL showed advanced hearing abilities compared to specimens of group XS and all specimens of groups S – XL were able to detect frequencies up to 6 kHz. The lowest threshold from 50 Hz to 2 kHz of all size groups (81.6 dB re 1 µPa at 1 kHz) was found in group XL. At frequencies from 3–6 kHz, group S showed the highest sensitivity of all size groups (77.7 dB re 1 µPa at 4 kHz). The lowest hearing threshold of group M was 82.7 dB re 1 µPa and 4 kHz; the corresponding values of group L were 85 dB re 1 µPa at 3 kHz (Figure 4, Table 1). In groups with a fully developed chain of ossicles (S – XL) the most sensitive frequency decreased with size from 4 kHz in groups S and M, to 3 kHz in group L and to 1 kHz in group XL. Significant correlations between size and hearing thresholds existed at most frequencies tested. At lower frequencies (50 Hz to 1 kHz), larger animals showed significantly better hearing abilities, whereas at the highest frequencies (4, 5 and 6 kHz) an opposite trend was found: smaller animals had lower hearing thresholds. At 2 and 3 kHz, no correlations were evident (Figure 7). The higher hearing abilities of groups S – XL compared to those of group XS are a clear consequence of the development of the chain of Weberian ossicles.

## Discussion

### Morphological development of Weberian ossicles

Several studies have addressed the ontogenetic development of siluriforms including the development and homology of the Weberian apparatus (see [40] for a review). The present study for the first time discusses the development from a functional point of view.

The development of the Weberian apparatus in otophysan fish varies considerably. The first appearance and the sequence of appearance of the Weberian ossicles apparently vary between taxa.

In the zebrafish *Danio rerio* the first Weberian ossicle appears at approximately 5 mm standard length (SL) [18], [19]. Higgs et al. [38] observed the chain of ossicles in 7-mm-long (TL) zebrafish and mentioned ‘gaps’ between ossicles. This indicates that a complete, uninterrupted connection between the swimbladder and the inner ear is missing in the zebrafish at this stage.

In the goldfish, the Weberian ossicles appear first at 10 mm [13]. Interestingly, Rosen and Greenwood [14] found no indications of a Weberian ossicle in the small characiform pencilfish *Poecilobrycon* (now *Nannostomus*) *harrisoni* at 7 mm SL.

In the zebrafish and the two characiform species *Brycon erythropterus* (nowadays *B. cephalus*) and the piranha *Serrasalmus* sp., the differentiation of ossicles progresses from posterior to anterior [18], [19].

A number of investigations in catfishes show that the claustrum appears last (*Silurus asotus* – Ichiyangi et al. [23]; *Pseudobagrus ichikawai* – Ichiyanagi et al. [24]; *I. punctatus* – Grande and Shardo [26]). In *L. cyclurus* the intercalarium is the last of the four ossicles to appear. This is in contrast to the observations mentioned above and to the observations of authors studying Weberian ossicles in catfishes having an intercalarium (in silurids [23] and ictalurids [25]).

Other studies fail to mention the sequence of ossicle appearance but do describe the appearance of all elements at a particular fish size. In the hardhead sea catfish *Galeichthys felis* the tripus, intercalarium, scaphium and the interossicular ligaments are developed at 14 mm total length (TL) (this species lacks a claustrum), but a “continuous stretch of tissue” between the bladder and inner ear is not yet developed at this stage [21]. In *Clarias gariepinnus* the Weberian ossicles are fully developed at 15 mm TL [22]. In *Corydoras paleatus*, which possesses only a single ossicle, this ossicle was found at 5–7.6 mm standard length (SL), but not fully ossified at this stage [25]. In ictalurids, ossification was observed at stages larger than 11.1 mm (SL) in *Ictalurus punctatus* and larger than 8.3 mm in the genus *Noturus* [25]. We cannot determine exactly at which size the chain of ossicles is fully developed in *Lophiobagrus cyclurus*. According to our morphological and physiological data a fully developed ossicular chain including interossicular ligaments is present in group S (min. 24.0 mm SL).

The right tripus of *Lophiobagrus cyclurus* is not a single ossicle (like the left tripus) at the earliest analysed stage (group XS) but is bipartite, a situation similar to that described in the goldfish by Watson [13]. This is because the tripus derives from different sources (parapophysis and pleural rib of the third vertebra, the former giving rise to the articulating process, the latter to the transformator process). These different origins were discussed in ictalurids by Coburn and Grubach [25] and in the cyprinid *Scardinius erythrophthalmus* by Matveiev [12] (see also Dahdul et al. [4] for a review on the development of the Weberian ossicles).

Most catfish in which an intercalarium is present show reduced or missing processes of the intercalarium [9]. The intercalarium of adult *L. cyclurus* also shows only a slight indication of a processus. This was described by Bornbusch [41] as “condition 0” in silurids: interossicular portion compressed, with a slender ascending process extending posterodorsally beyond the interossicular ligament.

The ossification of Weberian ossicles has been discussed in several papers: Watson [13] and Kulshrestha [15] mentioned that the intercalarium (manubrium) is an ossification of the interossicular ligaments in two cypriniform species. We suppose that the intercalarium has a similar origin in *L. cyclurus*. Grande and Shardo [26] found a full ossification of all ossicles at a size of about 15 mm in *Ictalurus punctatus*. In our study species *L. cyclurus*, specimens of the smallest size group XS (size 11.3–15.3 mm SL) did not yet have fully ossified ossicles. Our µCt scans showed only the small ossified parts of the ossicles and no cartilaginous areas, in contrast to the 3D reconstructions of the sections which showed ossified and cartilaginous parts. In the bagrid catfish *Pseudobagrus* (*Coreobagrus*) *ichikaway* the scaphium was ossified at 14.8 mm SL and all ossicles of the Weberian system at 25 mm SL [24]. This coincides with our observations and findings in specimens of groups XS and S of *L. cyclurus*, which show ossified ossicles and well-developed hearing beginning with stage S. We suppose that *P. ichikaway* possesses well-developed hearing capabilities, similar to *L. cyclurus*, at this stage.

### Development of auditory sensitivities

The present study reveals a relationship between hearing acuity and the development of the Weberian apparatus. Specimens of the smallest stage lacked a fully developed chain of Weberian ossicles and interossicular ligaments, suggesting that transmission of swimbladder oscillations to the inner ear is probably reduced or not yet possible. This may have affected absolute hearing thresholds and reduced the ability to detect higher frequencies (3–6 kHz). Surprisingly, the lack of a complete chain of Weberian ossicles had quite different effects in the zebrafish [38]. Those authors showed that the development of Weberian ossicles affected the bandwidth of detectable frequencies rather than absolute thresholds. The youngest zebrafish detected sounds up to 200 Hz (versus 2 kHz in our catfish), while older zebrafish detected frequencies up to 4 kHz. In contrast, we found that hearing sensitivity in *L. cyclurus* changed significantly at all frequencies. At this point we cannot explain the different results in zebrafish and *L. cyclurus*.

Our data support previous findings in the squeaker catfish *Synodontis schoutedeni* [39]. Within comparable size stages (>21 mm to adult), smaller specimens of both catfish species show better high-frequency hearing (4–6 kHz in *L. cyclurus* and 5–6 kHz in *S. schoutedeni*), and larger specimens show better low-frequency hearing (50 Hz–1 kHz). These changes are probably not caused by the development of the ossicular chain (which is already fully developed in *L. cyclurus* specimens of this size and should be also fully developed in similar sized *S. schoutedeni*), but by further, still unknown reasons.

Grande and Young [19] stated that the Weberian ossicles in 6.6–14.5 mm zebrafish are “in position to receive sound vibrations”. Higgs et al. [38] found “large gaps between individual ossicles” up to 13 mm TL in zebrafish and an unbroken chain of ossicles at 19.5 mm TL. They argue that the change in hearing is driven by the development of auxiliary specializations. This agrees with our findings namely that the Weberian apparatus is not fully developed in the smallest stage and that the maximum detectable frequencies (2–3 kHz) are lower than in later stages. However, both studies did not mention at which stages interossicular ligaments were present. Perhaps this explains partly why the smallest zebrafish did not respond to frequencies higher than 200 Hz. Higgs et al. [38] discussed this issue and mentioned the possibility that the extension of the frequency range could be due to the addition of saccular and lagenar hair cells, and to a significant increase in the perimeter of both saccular regions. They also argued that it is unlikely that the changes in the maximum detectable frequency are due to selective addition of high-frequency hair cells in the sacculus.

In an earlier study on the goldfish, Popper [35] detected no significant differences in hearing capabilities between fish of 5 and 10 cm SL. Popper's results can probably be explained by the fact that the major improvements in hearing take place in stages <20 mm and that his specimens were larger. Nevertheless, that study shows that larger fish, which have larger inner ear sensory maculae and thus more hair cells, do not hear better.

Generally, changes in hearing abilities during ontogeny are known in several fish species [27], [28], [29], [31], [32], [33]. None of these studies, however, examined the changes in the auditory periphery or the inner ear, preventing conclusions on which factors are responsible for the changes in hearing. Only Higgs et al. [30] discussed that structural changes in the utriculus might be correlated to the ability to detect ultrasound in the American shad *Alosa sapidissima*.

The present study supports recent findings in the squeaker catfish *S. schoutedeni* [39], where the first evidence of a change in auditory thresholds during ontogeny in an otophysine fish species has been reported. The sensitivity increase with size at the low and mid-frequency range agrees with findings in *S. schoutedeni* and non-otophysine fish.

The importance of the uninterrupted Weberian chain has been studied in adult otophysines by Poggendorf [42] and Ladich and Wysocki [43] by tripus extirpation in a catfish and a cyprinid, respectively. Poggendorf [42] showed that bilateral removal of the tripus in the ictalurid *Ameiurus nebulosus* resulted in a frequency-independent decrease in hearing sensitivity. In contrast, Ladich and Wysocki [43] showed that bilateral extirpation in the goldfish led to a hearing loss which increased with frequency and furthermore resulted in a narrower detectable frequency range. This frequency-dependency is more similar to our observations in *L. cyclurus* and supports our interpretation that hearing improvement depends on the presence of an uninterrupted Weberian chain of ossicles.

In summary, our study on the catfish *Lophiobagrus cyclurus* shows that the improved hearing abilities in this siluriform fish and probably in all otophysines depend on the development of the Weberian apparatus. We show that the freshly hatched catfish do not yet possess a fully developed Weberian apparatus, which probably reduces hearing acuity and the range of detectable frequencies. We furthermore illustrate that pronounced differences exist between the zebrafish and *L. cyclurus.*

## Materials and Methods

### Animals

All *Lophiobagrus cyclurus* (Worthington and Ricardo, 1937) were aquarium bred and obtained from Oliver Drescher (Vienna, Austria). Fish were kept in planted aquaria with a sand bottom equipped with roots and clay or bamboo tubes as shelters. In order to provide a quiet environment, we used only external filters and no internal filters or air stones. Temperature was kept at 25±1°C and a 12 h : 12 h L : D cycle was maintained. Fish were fed frozen chironomid larvae and artificial food (granulate, flakes and tablets); the small specimens of groups XS were also fed Cyclop-Eeze® (freeze-dried copepods, Argent Chemical Laboratories, Redmond, WA, USA). Since fry and juveniles grow very unequally despite identical conditions of husbandry [36], [44], we classified the tested specimens as different size groups rather than age groups, because size is more highly correlated with stage of osteological development than age [18]. Standard length (SL) was measured as “standard length 2” following Holcik et al. [45]. Using total length or body mass instead of SL for analyses did not change the results.

For hearing measurements, fish were grouped into five size groups, XS (SL = 11.3–15.3 mm, 0.04–0.08 g, N = 8), S (SL = 24.0–34.7 mm, 0.33–0.69 g, N = 10), M (SL = 39.0–47.8 mm, 1.52–2.82 g, N = 7), L (SL = 54.5–66.3 mm, 4.60–7.85 g, N = 8) and XL (SL = 68.9–83.9 mm, 8.43–15.8 g, N = 7). Complete audiograms could not be obtained from each fish, in particular in the smallest size group. A minimum of six hearing thresholds was determined for each group and each frequency.

### Examination of Weberian ossicles

For morphological examinations, fish were sedated using an overdose of tricaine methanesulphonate (MS-222, Sandoz, Basel, Switzerland) and then immediately preserved in alcohol (70%), formalin (4%) or Bouin's solution [46].

Dissections were carried out in alcohol-preserved specimens (of groups S, M, L and XL) with the aid of dissection microscopes (Wild M5, Wild Heerbrugg Ltd, Heerbrugg, Switzerland, and Nikon SMZ1500, Nikon Corporation, Tokyo, Japan). Bones and ossicles were stained in a solution of KOH (1%) and alicarin red. The photo of Weberian ossicles was taken using a Leica MZ16 F microscope (Leica Microsystems, Wetzlar, Germany) and a ProgRes C5 camera (Jenoptik, Jena, Germany); the photograph was edited with Adobe Photoshop CS5 (Adobe, San Jose, CA, USA).

Microcomputed tomography (µCT35, SCANCO Medical AG, Brüttisellen, Switzerland) was used to assess bone architecture using 3.5, 6, 10 and 15 µm isotropic voxel size (depending on the size of the scanned specimen). One specimen each of group XS, S, L and XL was scanned in 70% ethanol along the coronal axis. Images were acquired at 70 kV and 57 µA with a 0.18° rotation between frames. CT images were reconstructed in 2048×2048 pixel matrices using a standard convolution-backprojection procedure. The resulting greyscale image stack was cropped to the region of interest before importing it into 3D-reconstruction software Amira 4.1 (Mercury Computer Systems, Chelmsford, MA, USA). Bony structures were visualized as volume rendering using the Voltex tool and the VolrenGlow colormap of Amira. To accentuate the Weberian ossicles within the volume rendering, they were surface-reconstructed by labelling them with the magic wand tool of the Amira segmentation editor, with minor corrections conducted with the brush tool. A surface for each ossicle was created using Amira's SurfaceGen. Surfaces were optimised by iterated simplification and smoothing steps. Snapshots of the surface reconstruction were taken with Amira.

For paraffin-based histology, three animals of stage XS were fixed in Bouin's solution [46] for 30 days, changing the solution twice a week. After complete fixation and decalcification, the samples were dehydrated in a graded ethanol-isopropanol series and embedded in paraffin. After polymerisation, 7-µm serial-sections were made on a Reichert-Jung 2030 rotary microtome (Reichert-Jung, Bensheim, Germany). The sections were mounted on glass slides and, after removing the paraffin, standard stained with Haematoxylin-Eosin (H-E) and Azan (after [46], [47]). Stained sections were analysed and documented by digital photography under a Nikon Eclipse E800 light microscope equipped with a Nikon DS-5MU1 digital camera (Nikon Corporation, Tokyo, Japan).

One specimen of group XS was decalcified for resin-embedding by storage in Bouin's solution for several weeks. After decalcification, the specimen was transferred to 70% alcohol, dehydrated in graded alcohol, and embedded in Agar low viscosity resin (Agar Scientific, Stansted, England) using acetone as intermediate. Ribbons of serial sections (2 µm section thickness) were obtained with a Histo Jumbo diamond knife on a Reichert Ultracut S microtome (Reichert-Jung, Bensheim, Germany) [48]. Sections were stained with Toluidine blue and photomicrographs were captured as described above for histological sections. Images were reduced in size and converted to greyscales using Adobe Photoshop CS3 (Adobe, San Jose, CA, USA) before importing the image stack into Amira 4.1. The image stack was aligned with the AlignSlices tool of Amira. Structures, i.e. the Weberian ossicles, central nervous system, inner ear, chorda dorsalis and the first neural arch were labelled manually with a brush before creating a surface for each structure. Surface generation and optimization was conducted as mentioned for the reconstruction of ct-scanned specimens, and snapshots were taken with the Amira software.

### Auditory sensitivity measurements

Hearing thresholds were obtained using the AEP recording technique developed by Kenyon et al. [49] and modified by Wysocki and Ladich [50], [51]. Only a brief description of the technique is given here. Test fish of groups S - XL were mildly immobilized with Flaxedil (gallamine triethiodide; Sigma-Aldrich, Vienna, Austria) diluted in a Ringer solution. The dosage applied (0.3–1.81 µg g-1) allowed fish to still perform slight opercular movements but not to initiate significant myogenic noise that could interfere with the AEP recordings. Specimens of group XS were not immobilized because of their small size. All auditory measurements were carried out in a bowl-shaped plastic tub (diameter 33 cm, water depth 13 cm, 1 cm layer of gravel), which was lined inside with acoustically absorbent material (air-filled packing wrap) to decrease resonances and reflections [52]. The tub was positioned on an air table (TMC Micro-g 63–540, Technical Manufacturing Corporation, Peabody, MA, USA), which rested on a vibration-isolated plate of concrete. A sound-proof chamber, constructed as a Faraday cage (interior dimensions: 3.2 m×3.2 m×2.4 m), enclosed the whole setup. The subjects were placed at the water surface in the center of the tub. The contacting points of the electrodes were maximally 1–2 mm above the water surface. Tissue paper (Kimwipes®) was placed on the fish head to keep it moist and ensure proper contact of electrodes. Respiration was achieved through a temperature-controlled (25±1°C), gravity-fed water circulation system using a pipette inserted into the animal's mouth. The AEPs were recorded by using silver wire electrodes (0.38 mm diameter) pressed firmly against the skin: the recording electrode was placed over the region of the medulla and the reference electrode cranially between the nares. Shielded electrode leads were attached to the differential input of an AC preamplifier (Grass P-55, gain 100×, high-pass at 30 Hz, low-pass at 1 kHz), with a ground electrode placed in the water near the fish's body. A hydrophone (Brüel and Kjaer 8101, Naerum, Denmark; frequency range 1 Hz to 80 kHz±2 dB; voltage sensitivity −184 dB re 1 V µPa^−1^) was placed close to the head on the right side of the animals (∼1 cm away) in order to determine absolute stimulus SPLs. A custom-built preamplifier was used to boost the hydrophone signal. Both presentation of sound stimuli and AEP waveform recording were achieved using a modular rack-mount system [Tucker-Davis Technologies (TDT) System 3, Gainesville, FL, USA] controlled by a PC containing a TDT digital signal processing board and running TDT BioSig RP software.

### Presentation of sound stimuli

Hearing thresholds were determined at 0.05, 0.07, 0.1, 0.3, 0.5, 0.8, 1, 2, 3, 4, 5 and 6 kHz. The duration of sound stimuli increased from two cycles at 50 Hz, 70 Hz and 100 Hz up to eight cycles at 4 kHz and above. Rise and fall times increased from one cycle at 50 to 300 Hz, up to three cycles at frequencies from 2 to 6 kHz. All bursts were gated using a Blackman window. For each test condition, one thousand stimuli were presented at opposite polarities, i.e. 90° and 270°, and were averaged together by the BioSig RP Software, yielding a 2000-stimulus trace to eliminate any stimulus artifact. The SPL was reduced in 4-dB steps. Close to hearing threshold, this procedure was performed twice and the AEP traces were overlaid to visually check if they were repeatable. The lowest SPL at which a repeatable AEP trace could be obtained, as determined by overlaying replicate traces, was defined as the threshold (see also [53]). Sound stimuli waveforms were created using TDT SigGen RP software. Tone-bursts were presented through two speakers (Fostex 256 PM-0.5 Sub and PM-0.5 MKII, Fostex Corporation, Tokyo, Japan). These were positioned 0.5 m above the water surface.

### Statistical analyses

All data were tested for normal distribution using Shapiro-Wilk's test. When data were normally distributed, parametric statistical tests were applied. Mean hearing thresholds were determined for each size group and at each frequency, and audiograms were drawn using SigmaPlot 10.0 (Systat Software/Cranes Software Inc., Bangalore, India and San Jose, USA). Means of sound characteristics were calculated for each fish and used for further analyses. Audiograms of different size groups were compared by a two-factor analysis of variance (ANOVA) using a general linear model where one factor was size group and the other was frequency. A Scheffé's post hoc test revealed which groups differed from each other. Relationships between fish size (SL) and hearing thresholds were determined by Pearson's correlation coefficients and linear regressions. The statistical tests were performed with the software SPSS 17.0 (SPSS Inc., Chicago, Illinois). When datapoints showed two different distribution patterns, segmented linear regressions and breakpoints were calculated using the software SegReg (R. J. Oosterbaan, Wageningen, The Netherlands).

The study protocol was approved by the Austrian Federal Ministry of Science and Research, permit number GZ 66.006/0023-II/10b/2008.
